# Effect of Pulp Chamber Access, Instrumentation, Obturation, and Restoration on the Fracture Resistance of Endodontically Treated Canine Teeth in Dogs

**DOI:** 10.1177/08987564241264036

**Published:** 2024-07-23

**Authors:** Maya Alexandra Popovic, Bertrand Lussier, Kambiz Chizari, Yvan Dumais

**Affiliations:** 1 Centre Hospitalier Universitaire Vétérinaire, Faculty of Veterinary Medicine, 5622 University of Montreal, St-Hyacinthe, QC, Canada; 2 Department of Clinical Sciences, Faculty of Veterinary Medicine, 5622 University of Montreal, St-Hyacinthe, QC, Canada; 3 Department of Mechanical Engineering, 5596 Polytechnique Montreal, Montreal, QC, Canada

**Keywords:** tooth fracture, endodontic treatment, canine tooth, veterinary dentistry, fracture resistance, force to fracture

## Abstract

Veterinary studies documenting the effect of endodontic treatment on tooth fracture resistance are scarce. The objective of this ex vivo study was to evaluate the effects of mesial access preparation and restoration, as well as pulp chamber access, instrumentation, obturation, and restoration, on the fracture resistance and characteristics of canine teeth in dogs. Sixty-five dog canine teeth were divided into 4 groups: 1. Standard endodontic treatment through a mesial access only; 2. Treatment as per group 1, adding an incisal access, instrumentation and obturation of the pulp chamber, and restoration of the access; 3. Treatment as per group 2, without pulp chamber obturation or restoration of the incisal access; and 4. Untreated teeth. The fracture resistance and characteristics of each group were documented using axial compression testing, angled 45° disto-occlusal to the long axis of the crown. The maximum force prior to fracture in groups 1, 3, and 4 were not statistically different, demonstrating that restored mesial and incisal accesses with pulp chamber instrumentation did not statistically affect fracture resistance. However, obturated and restored group 2 teeth demonstrated decreased fracture resistance compared to all other groups (*P* < .001). Additionally, 26.7% of group 1 teeth sustained complicated crown fractures, while 100% of group 2 teeth fractured within the obturation or restorative materials, preventing pulp exposure in these cases. Although the cause and clinical importance of decreased tooth fracture resistance following pulp chamber obturation and restoration remains unknown, it may provide protective value for maintaining a coronal seal in the event of tooth fracture.

## Introduction

Endodontic disease, defined as infection or inflammation of the tooth pulp, is a common disorder in canine patients and is often a consequence of traumatic dentoalveolar injuries (TDI).^1^ In dogs, tooth fracture is the most common TDI reported,^2^ affecting more than one-quarter of the general population.^3^ Enamel–dentin–pulp and deep enamel–dentin fractures result in pulp and near-pulp exposure, respectively, leading to microbial infection of the endodontic system.^4^ Pulpitis and pulp necrosis ensue, which progresses to inflammation of the periapical tissues, known as apical periodontitis.^5^

Following tooth fracture, concussion is the second-most prevalent TDI, representing 14.4% of all TDI in one study.^2^ Severe concussion clinically manifests as tooth discoloration due to pulpitis, or inflammation of the pulpal tissues. Clinical studies in dogs demonstrate that approximately 90% of discolored teeth have pulp necrosis, necessitating treatment.^6,7^

Treatment options for endodontically compromised teeth include either root canal therapy or extraction. Standard endodontic therapy, or root canal therapy, is the treatment of choice as it allows for a minimally invasive treatment approach compared to tooth extraction. It also preserves tooth structure and function, and has a high success rate reported in multiple published clinical reports.^1,8-10^ This is especially important for canine and carnassial teeth, which account for over 60% of all TDI in dogs.^2^

There are several published protocols for standard endodontic therapy in dogs.^1,11-15^ This is achieved through access, instrumentation, disinfection, and obturation (sealing) of the endodontic system, followed by restoration of the access site.^4^ In human dentistry, long term clinical success of endodontic treatment is not only impacted by resolution of infection and inflammation, but also by the preservation of sound tooth structure and fracture resistance.^16^ Although each step of endodontic treatment can negatively impact tooth fracture resistance, excessive tooth loss from root canal access and restorative methods presents a major concern in human endodontics. This has led to the development of several conservative access preparations, in order to prevent the formation of a large cavity resulting from more invasive traditional accesses.^17^

In dogs, bite forces at the level of the canine tooth are demonstrated to range from 147 to 926 N,^18,19^ exceeding the fracture resistance of their canine teeth.^20,21^ This may have important clinical implications for endodontic treatment success, thus demonstrating the importance of further research in the mechanical effects of endodontic treatment in dogs. However, veterinary studies investigating the effects of different endodontic treatment approaches on tooth fracture resistance are lacking. To date, only one study exists on the effect of endodontic treatment on the fracture resistance of dog teeth. It was performed on maxillary fourth premolar teeth and demonstrated no significant difference between different root canal access methods.^22^ Given that the canine teeth are implicated in 35–40% of all TDI in dogs and also account for a majority of endodontic treatments in veterinary dentistry,^2,3,9,10^ the objective of this study was three-fold. The first objective was to evaluate the effect of standard endodontic treatment on canine teeth in dogs by observing the effect of mesial access preparation and restoration on the fracture resistance of endodontically treated dog canine teeth. The second objective was to observe the effect of pulp chamber access, instrumentation, obturation, and restoration on fracture resistance of endodontically treated dog canine teeth. The third objective of this study was to document any differences in fracture characteristics between test groups. The authors hypothesized that there would be no significant difference in fracture resistance or characteristics of endodontically treated canine teeth of dogs, by using an axial compression ex vivo model.

## Materials and Methods

### Specimen Collection

Twenty mesocephalic small- to medium-breed dog heads with intact canine teeth were selected from fresh–frozen cadavers previously euthanized for reasons unrelated to this study. Any teeth with evidence of tooth fracture or abrasion were excluded. Eighty canine teeth were harvested via surgical extraction. Crown integrity was confirmed by visual inspection and lateral radiographs following extraction. Any tooth with radiographic evidence of pulp chamber or root canal obliteration was excluded. To select teeth of similar size, the hard tissue volume of each tooth was calculated as previously described.^20^ The total crown volume was calculated by using calipers to measure the crown height, major base diameter, and minor base diameter, then these values were placed in the formula for a cone (*V* = 1/3(*πr*^2^*h*)). Next, the pulp chamber volume was calculated by radiographic measurement of the pulp chamber height and diameter, using the volume of a cone for subjectively younger blunderbuss canals, or the volume of a cylinder for subjectively older cylindric canals (*V* = *πr*^2^*h*). The total hard tissue volume in the crown was subsequently determined by deducting the pulp chamber volume from the total crown volume. Teeth within 40% of the calculated median hard tissue volume were included. A total of 65 teeth met all inclusion criteria. The teeth were then fixed in 10% formalin for a minimum of 5 days prior to storage in room-temperature distilled water.^23^

### Group Allocation

Pre-statistical analysis was performed to confirm homogeneity between groups. Teeth were randomly allocated into four treatment groups using statistical software^a^, with equal distribution of maxillary and mandibular canine teeth into each group. Given positive correlations between hard tissue volume and height to diameter (H/D) ratio on fracture resistance,^20^ ANOVA testing was used to confirm homogeneity between groups for these two variables (*P *= .919 and *P *= .492, respectively). In addition, homogeneity was also confirmed for pulp chamber volume via Kruskal–Wallis testing (*P *= .910).

### Specimen Preparation

**Figure 1.**
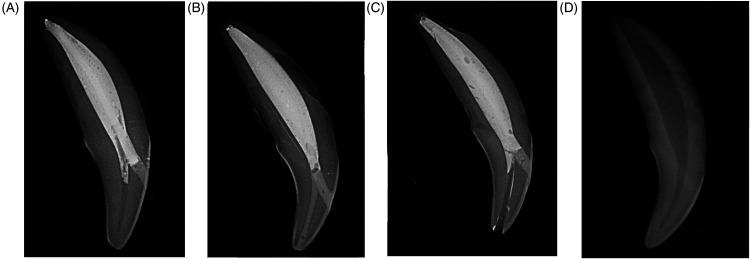
Radiographs of the 4 treatment groups. (A) Group 1: Standard endodontic treatment through a mesial access only with root canal obturation followed by restoration of the mesial access site using resin-modified glass ionomer (RMGIC) and composite resin. The pulp chamber was left untreated. (B) Group 2: Standard endodontic treatment through mesial and incisal access sites. The pulp chamber was obturated with RMGIC prior to routine root canal obturation. Then, both access sites were restored with composite resin. (C) Group 3 (negative control): Teeth were subjected to the same treatment as group 2, without obturation or restoration of the pulp chamber and incisal access site, respectively. (D) Group 4 (positive control): Intact, untreated tooth.

Teeth were removed from distilled water storage and prepared according to one of four randomly assigned treatment groups (Figure 1). To test the effect of mesial access and restoration on tooth fracture resistance, group 1 teeth (*n* = 16) were treated with standard endodontic therapy using a mesial access only, without treatment of the pulp chamber. A mesial access to the root canal measuring 2 to 3 mm in diameter was made with a #3 round bur on a high-speed dental hand piece, 3 to 4 mm coronal to the cementoenamel junction (CEJ). Following access, the root canal was filed, shaped, and cleaned in a standard fashion using lubricated^b^ hand and rotary files.^1,11^ During instrumentation, 3% sodium hypochlorite and 0.9% saline were used to irrigate the canals. After removing the smear layer from the prepared root canal with 17% ethylenediaminetetraacetic acid (EDTA)^c^ for 15 seconds, a final disinfection was performed with 3% sodium hypochlorite before a final rinse with 0.9% saline. Paper points were used to dry the canal prior to obturation with a cold-flowable gutta percha obturation material^d^ and a single gutta percha master cone^e^. Access site restoration was performed using a resin-modified glass ionomer (RMGIC)^f^ intermediate layer, etching the cavity with 37% phosphoric acid before application of a 5^th^ generation bonding agent^g^ and final composite restoration^h^. The final restoration was smoothed with an Arkansas stone and post-operative radiographs were performed to document any voids in obturation or restoration.

**Figure 2.**
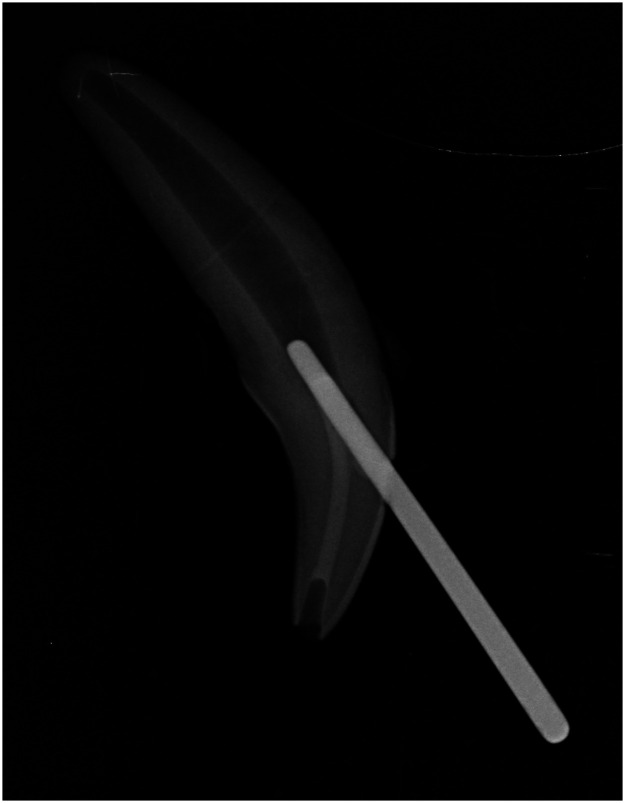
Radiograph of the resin-modified glass ionomer (RMGIC) obturation of the pulp chamber. A large gutta percha cone was placed in the mesial access site of the tooth to prevent leakage of the pulp chamber obturation material into the mesial access site and root canal. The gutta percha cone was removed prior to photopolymerization of the RMGIC and completion of the endodontic treatment.

To test the effect of pulp chamber obturation and restoration on the fracture resistance of dog canine teeth, group 2 teeth (*n* = 17) were treated as per group 1, with the addition of an incisal access for instrumentation and obturation of the entire pulp chamber. A #1 round bur was used to create an access at the incisal surface of the canine crown. The pulp chamber was instrumented with Hedstrom files used sequentially, by increasing the size of the file until size #50. To prevent the pulp chamber from being filled with the material used for obturation of the root canal, it was obturated first. This was achieved by placing a large gutta percha point in the mesial access to block the opening into the root canal and allow exclusive obturation of the pulp chamber with RMGIC through the incisal access (Figure 2). A space of approximately 2 mm was left in the incisal access to allow space for the final restoration before photopolymerization for 30 seconds on each side. The gutta percha point was then removed from the mesial access site. The root canal was obturated and restoration of both access sites was performed as previously described.

To test specifically the effect of pulp chamber access and instrumentation, group 3 teeth (negative control, *n* = 16) were prepared as per group 2, except that the pulp chamber and incisal access site were not obturated nor restored respectively, therefore leaving the pulp chamber and incisal access empty.

To serve as a positive control, group 4 teeth were not treated and were left intact (positive control, *n* = 16).

Following sample preparation and before mechanical testing, all teeth were stored at room temperature in distilled water for a minimum of 3 weeks and a maximum of 6 months. This timeframe was selected to allow for optimal polymerization of the RMGIC obturation material.^23-28^ During the storage period, teeth were potted into rectangular silicone molds filled with polymethyl-methacrylate (PMMA)^i^ to a point 1 to 2 mm apical to and parallel to the CEJ. The PMMA was allowed to cure for approximately 30 minutes prior to returning to distilled water storage.

**Figure 3.**
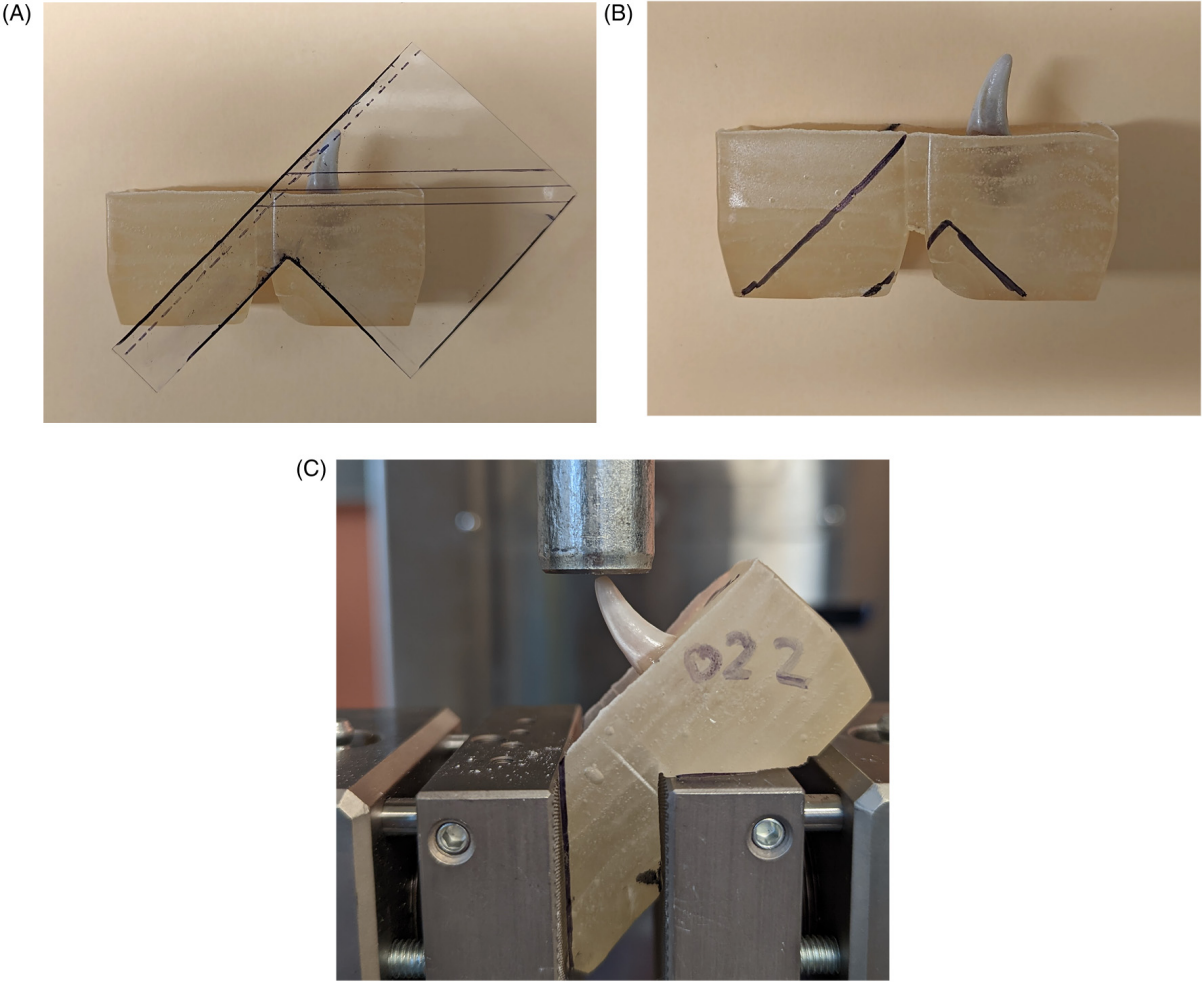
Polymethyl-methacrylate (PMMA) block preparation and mechanical testing set-up. A plastic cutting guide was fabricated such that when aligned to the cemento-enamel junction (CEJ) of the tooth, the outlined model would fit within a screw action grip attachment of a universal testing machine (UTM) with a consistent 45° angle relative to the long axis of the crown. (A) The cutting guide applied to a test sample, aligning with the CEJ. (B) Outline of the cutting guide, which was then used for making cuts using an oscillating saw. (C) Representative sample mounted in a UTM, demonstrating application of the load on the incisal surface at a 45° angle relative to the long axis of the crown.

To allow the samples to fit within the attachment grips during mechanical testing, a custom cutting guide was fabricated to cut the PMMA blocks (Figure 3). This also served to standardize the force application, 45⁰ disto-mesial to the long axis of the crown at the incisal surface. This angle was selected to allow application of both compressive and sheer forces, thus simulating biting and pulling forces in dogs, respectively.^20^ To prepare each sample, potted teeth were briefly removed from distilled water storage and dried. The cutting guide was precisely aligned to the CEJ of the tooth and its edges outlined on the PMMA blocks with permanent marker. Then, the PMMA was trimmed and smoothed with an oscillating saw and belt sander, respectively. The samples were then returned to room temperature distilled water storage until mechanical testing.

### Mechanical Testing

Mechanical testing was adapted from previously described protocols.^20-22^ All prepared samples were photographed prior to testing. Samples were randomly selected to undergo continuous axial compression testing using a screw action grip attachment^j^ on a universal testing machine^k^ (UTM) equipped with a 1-kN load cell^l^ and a 19-mm diameter stainless steel rod for force application (Figure 3C). Pre-testing photographs were obtained to determine the force angle applied to each sample which was calculated using imaging software^m^.^22^ The testing protocol was initiated using a crosshead speed of 1 mm/min, in a disto-mesial direction 45° to the long axis of the crown, starting approximately 0.5 mm away from the incisal surface. Force/displacement curves were recorded^n^ until failure, which was defined as an instantaneous decrease of force >50%. The maximum force prior to fracture in Newtons (N) was documented. Post-testing photographs and radiographs were performed to interpret fracture type adapted from the American Veterinary Dental College (AVDC) classification system of complicated versus uncomplicated fracture types, where a breach in coronal seal via pulp exposure, or complete loss of obturation/restorative materials from the pulp chamber, were defined as complicated fracture.^29^

### Statistical Analysis

Statistical analysis was performed using R^a^ with 95% confidence intervals and reporting significance when *P* < .05. Homogeneous and normally distributed variables were reported as means (+/− standard deviation), whereas variables not fulfilling these conditions were presented as medians (interquartile range, IQR 1-IQR 3).

To select appropriate statistical tests for comparing treatment groups, dependent variables were tested for normality and homoscedasticity using Shapiro–Wilks and Bartlett tests, respectively. Total hard tissue volume, impact angle, stiffness, H/D, pulp length, and tooth volume fulfilled these conditions, so they were compared between groups using linear models. Kruskal–Wallis analysis was used to compare variables not fulfilling these conditions, including maximum force prior to fracture, crown height, pulp volume, major base diameter, minor base diameter, and pulp diameter. A generalized linear model was used to analyze pulp shape given its binomial nature. For any dependent variables demonstrating statistical differences between treatment groups, post-hoc testing was used to determine which groups differed, while applying necessary corrections to the *P*-values using the Benjamin–Hochberg method. Finally, to test for an association between fracture types and treatment group, Chi-squared (*χ*^2^) tests were used.

## Results

**Figure 4.**
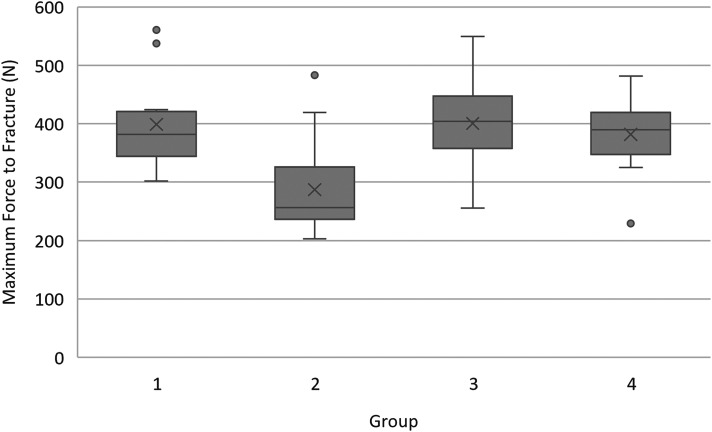
Box and Whisker plots of maximum force prior to fracture for each treatment group. Group 2 teeth, where the pulp chamber was obturated and restored, were significantly weaker than all other groups (*P* < .001). No significant difference was seen between groups 1, 3, and 4. Abbreviations: Circle = outlier. X = mean. Grey box = IQR, with the line in each box representing median. Whiskers = minimum and maximum values.

A total of 63 teeth were evaluated. One tooth in group 4 was excluded due to accidental dehydration and a second in group 3 due to the PMMA yielding before the tooth during mechanical testing. Statistical comparisons of structural and loading variables between groups are represented in Table 1, with the maximum force sustained prior to fracture for each tooth represented in Figure 4.

**Table 1.** Mean With Standard Deviations (SD) or Median With Interquartile Ranges (Q1–Q3) of Structural and Loading Variables, With Between-Group Comparisons.

	Group 1	Group 2	Group 3	Group 4	*P*-Value
Major base diameter (*D*) (mm)	8.0 (7.4–9.0)	8.0 (7.00–8.5)	8.0 (7.0–8.8)	8.0 (7.6–8.5)	.968
Minor base diameter (mm)	5.0 (5.0–5.0)	5.0 (5.0–5.0)	5.0 (5.0–5.0)	5.0 (5.0–5.0)	.437
Crown height (*H*) (mm)	15.0 (14.0–16.0)	14.5 (14.5–15.0)	15.0 (13.5–15.0)	15.0 (13.8–16.0)	.881
*H*/*D*	1.86 (0.19)	1.88 (0.17)	1.81 (0.20)	1.82 (0.15)	.734
Tooth volume (mm^3^)	160.7 (33.0)	152.4 (23.8)	154.3 (32.1)	155.5 (27.5)	.867
Pulp diameter (mm)	0.80 (0.70–1.13)	0.70 (0.60–1.80)	0.90 (0.60–1.75)	0.80 (0.70–1.40)	.891
Pulp height (mm)	10.19 (1.70)	10.32 (1.57)	9.89 (1.59)	9.82 (1.80)	.808
Pulp shape (cylinder/cone)	Cylinder 12 Cone 4	Cylinder 12 Cone 5	Cylinder 10 Cone 5	Cylinder 11 Cone 4	.961
Pulp volume (mm^3^)	5.08 (3.86–7.36)	3.73 (3.31–9.11)	6.04 (2.30–9.36)	5.08 (3.52–8.42)	.913
Total hard tissue volume (mm^3^)	152.94 (30.99)	145.32 (19.85)	147.45 (27.72)	148.01 (23.53)	.858
Impact angle (°)	43.1 (3.0)	43.6 (2.9)	44.5 (3.2)	44.2 (2.4)	.531
**Stiffness (N/mm)**	**599 (177)**	**639 (186)**	**442 (112)**	**615 (157)**	**.011**
**Maximum force prior to fracture (N)**	**382 (345–415)**	**256 (240–322)**	**404 (360–435)**	**389 (357–417)**	**<.001**

When comparing maximum force prior to fracture, groups 1, 3, and 4 demonstrated similar median forces prior to fracture of 382 N (345–415 N), 404 N (360–435 N), and 389 N (357–417 N) respectively. Conversely, obturated and restored group 2 teeth demonstrated a median maximum force prior to fracture of 256 N (240–322 N). This difference was statistically significant (*P *< .001), with pair-wise comparisons demonstrating that obturated and restored group 2 teeth were significantly weaker than all other groups.

A total of five potential outliers were identified when evaluating for maximum force sustained prior to fracture. Three group 3 teeth fractured at approximately 1.5× greater than the group 3 median (538 N, 541 N, 561 N), one group 2 tooth at 1.9× greater than the group 2 median (483 N), and one group 4 tooth at 1.7× less than the group 4 median (229 N). All these teeth came from different specimens and were not statistically different from the remainder of teeth in any structural and loading variables. Outliers were thus included in all subsequent statistical analyses.

**Figure 5.**
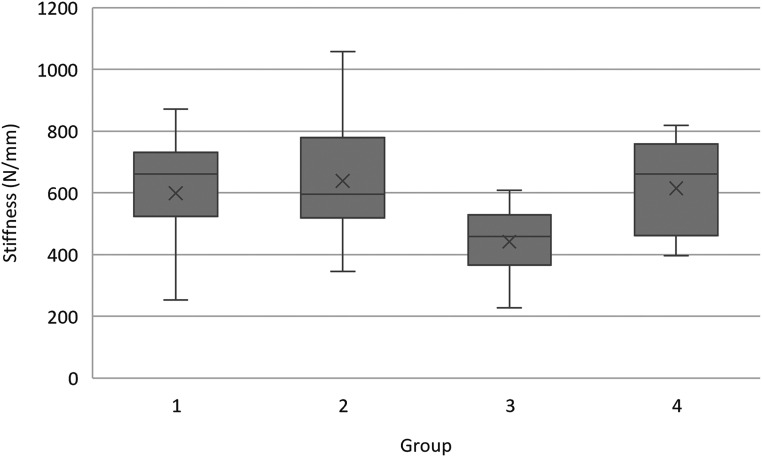
Box and Whisker plots of stiffness for each treatment group. Group 3 teeth, where the pulp chamber was instrumented but not obturated or restored, were significantly less stiff than all other groups (*P* < .011). No significant difference was seen between groups 1, 2, and 4. Abbreviations: X = mean. Grey box = IQR, with the line in each box representing median. Whiskers = minimum and maximum values.

In addition to maximum force prior to fracture, tooth stiffness (N/mm) between groups was determined to be significantly different (*P *< .011). This was estimated by calculating the slope of the force versus displacement curve for each tooth^30^ (Figure 5). Pair-wise comparisons demonstrated that group 3 teeth, which underwent incisal access and pulp chamber instrumentation without obturation and restoration, were significantly less stiff than groups 1, 2, and 4. No difference in stiffness was seen between groups 1, 2, and 4, and no outliers were identified in any group.

All structural variables were evaluated between groups (Table 1). No significant difference was seen in any structural variables, including major base diameter (*P *= .968), minor base diameter (*P *= .437), crown height (*P *= .881), H/D (*P *= .734), tooth volume (*P *= .867), pulp diameter (*P *= .891), pulp height (*P *= .808), pulp shape (*P *= .961), pulp volume (*P *= .913), and total hard tissue volume (*P *= .858). In addition, the mean impact angle during mechanical testing was calculated at 43.8° ± 2.9° and was not statistically different between groups (*P *= .531).

**Figure 6.**
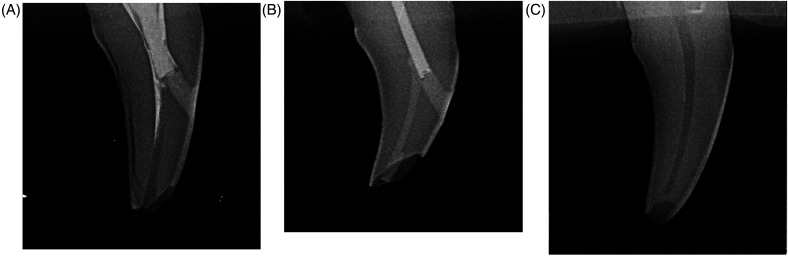
Radiographs of fracture types observed following mechanical testing of endodontically treated dog canine teeth, using a modified AVDC fracture classification system. (A) Complicated crown fracture with pulp exposure in a group 1 tooth, which underwent endodontic treatment with a mesial access only. In addition to pulp exposure secondary to an enamel–dentin–pulp fracture, a fissure is apparent coursing between the enamel and dentin layers of the distal part of the crown. (B) Uncomplicated crown fracture in a group 2 tooth, which underwent endodontic treatment with obturation and restoration of the pulp chamber and incisal access. This tooth was classified as an uncomplicated crown fracture, given the presence of obturation and restoration materials maintaining a coronal seal. (C) Uncomplicated crown fracture in a group 4, untreated tooth. There was no pulp exposure observed, since the fracture was isolated to the incisal tip of the crown.

Fracture characteristics of each tooth were evaluated visually and radiographically (Figure 6). All group 3 teeth were excluded from evaluation given that all had pulp exposure secondary to preparation technique, and one group 1 tooth was excluded due to a sustaining a crush injury following mechanical testing.

Overall, 11/15 (73.3%) of group 1 teeth sustained uncomplicated crown fractures, while 4/15 (26.7%) displayed complicated crown fractures (Table 2). 17/17 (100%) of group 2 teeth manifested fracture within the restorative or obturation material and were thus classified as uncomplicated crown fracture since a coronal seal was maintained. In group 4, 14/15 (93.3%) teeth sustained uncomplicated crown fracture, while 1/15 (6.7%) tooth displayed complicated crown fracture. There was no statistical difference when comparing group 1 versus group 2 (*P *= .082), group 1 versus group 3 (*P *= .327), or group 2 versus group 4 (*P* = .949). Finally, further evaluation of group 2 teeth revealed that most teeth manifested fracture at the incisal tip within the composite layer (13/17 teeth, 76.5%), while the remaining teeth sustained fractures at the junction of composite and RMGIC (1 tooth, 5.9%), in both the RMGIC and composite layers (2 teeth, 11.8%), or within the RMGIC (1 tooth, 5.9%).

**Table 2.** Frequency of Fracture Type in Each Treatment Group.

Group	Uncomplicated crown fracture	Complicated crown fracture	Total
1	11 (73.3%)	4 (26.7%)	15
2	17 (100%)	0 (0%)	17
3	–	–	–
4	14 (93.3%)	1 (6.7%)	15

## Discussion

This study is the first to report the effects of endodontic treatment on the fracture resistance of canine teeth in dogs. When compared to untreated group 4 teeth, there was no significant difference in fracture resistance found between group 1 teeth subjected to endodontic treatment with exclusively mesial access and restoration, or when adding incisal access and pulp chamber instrumentation as performed in group 3 teeth. These findings demonstrate that mesial access, incisal access, and pulp chamber instrumentation do not significantly affect tooth fracture resistance in dog canine teeth, when using the described ex vivo model. This is contrary to human studies where tooth weakening is observed secondary to endodontic access and restoration preparations, with preparation size inversely correlated with fracture resistance.^16,31-33^ The difference in this study may be explained by the relatively conservative access and instrumentation techniques employed, when compared to human preparation techniques.

Surprisingly, group 2 teeth subjected to obturation of the pulp chamber and restoration of the incisal access demonstrated a significant reduction in fracture resistance. This was unexpected, given that the addition of restorative materials typically improves fracture resistance when compared to unfilled controls, such as group 3 teeth in this study.^31-33^ Since group 3 teeth had similar fracture resistance to group 4 intact teeth, it was unlikely that the tooth weakening was secondary to incisal access and pulp chamber instrumentation, and was rather a consequence of the pulp chamber obturation and incisal access restoration. Restorative materials such as RMGIC and composite resin undergo physical alterations based on their chemical properties and handling conditions, which may in turn contribute to stress applied to endodontic access cavities. Examples of these physical alterations include polymerization-induced shrinkage stress,^33-37^ expansion stress from hygroscopic expansion,^37-40^ varying environmental conditions contributing to dimensional changes,^41,42^ and chemical degradation over time.^43^ In an access cavity, the interplay of these physical alterations may apply excess stress on the dentinal walls, inducing micro-fissure formation, crack propagation, and weakening of restored teeth. Future investigations are necessary to determine the cause of tooth weakening observed in this study, and whether or not other techniques or combinations of materials may have proven more effective in preserving fracture resistance.

Although elastic modulus would have provided a more accurate measurement of stiffness, variations in sample size coupled with complex tooth geometries rendered this calculation beyond the scope of this study. In evaluating stiffness using the slope of the force versus displacement curve for each tooth, no significant difference was seen between all treatment groups, except for group 3 teeth subjected to incisal access and instrumentation, without obturation and restoration of the pulp chamber. Group 3 teeth were significantly less stiff compared to all other groups, demonstrating that the removal of dental tissue at the incisal access and pulp chamber reduced tooth stiffness. Conversely, group 2 teeth had similar stiffness to group 1 and group 4 teeth, demonstrating that subsequent obturation and restoration re-established any loss in stiffness secondary to incisal access and pulp chamber instrumentation. These patterns have also been observed in human endodontic studies,^44,45^ with later studies demonstrating that access and restorative techniques affect tooth biomechanical properties, influencing strain, failure mode, and stress distribution.^46,47^ This has important clinical implications in endodontics, given that changes in tooth stiffness and elastic modulus may lead to unfavorable fracture patterns thereby increasing the incidence of untreatable fractures necessitating extraction. Thus, the goal of restorative techniques is not only to maintain tooth strength, but also tooth stiffness and biomechanics.

Evaluation of fracture type demonstrated that 100% of group 2 teeth with pulp chamber obturation and restoration sustained uncomplicated crown fracture which maintained a coronal seal, mostly within the composite restoration. A similar fracture location was seen in all other treatment groups, resulting in predominantly uncomplicated fractures at the incisal tip of the crown. No significant difference was seen in the frequency of complicated crown fractures between groups, affecting 26.7% of group 1 teeth, 0% of group 2 teeth, and 6.7% of group 4 teeth, but approached clinical significance when comparing group 1 versus group 2 teeth (*P *= .082). Conversely, one study demonstrated that greater than 75% of crown fractures in dogs result in pulp exposure.^2^ Therefore, this ex vivo study underestimates the severity of tooth fracture patterns seen clinically, where fractures are more likely to occur deeper than the incisal tip of the crown, such as in the middle and gingival thirds. As a result, the clinical importance of pulp chamber obturation and restoration becomes understated, where a vast majority of crown fractures would maintain a coronal seal in endodontically treated teeth (as performed in group 2 teeth). Conversely, the consequences of leaving the pulp chamber untreated were likely underestimated using an ex vivo design, where a new fracture would lead to pulp exposure in most clinical cases (as performed in group 1 teeth). This has important implications in veterinary patients, where delayed diagnosis of tooth fracture or repeat fracture is a common clinical scenario, and re-infection through root canal obturation and sealing materials may occur within days to weeks.^48-51^

In evaluating clinical situations benefiting from endodontic treatment with or without pulp chamber obturation and restoration, two main clinical scenarios apply: endodontic treatment of fractured versus unfractured teeth. Although decreased tooth fracture resistance was observed in group 2 teeth with obturated and restored pulp chambers, this may be less likely to significantly impact canine teeth presenting with crown fractures. These teeth sustain crown height loss which naturally decreases H/D, thus increasing fracture resistance.^22^ This in turn may mitigate possible tooth weakening from obturation and restorative techniques. Therefore, in clinical situations of fractured teeth undergoing endodontic treatment, pulp chamber obturation and restoration likely proves useful in optimizing endodontic treatment success, especially when subsequent fracture is a concern. Conversely, it remains unclear whether performing endodontic treatment of the pulp chamber is the optimal approach for intact teeth. Although the results of this study suggest that conservative endodontic access and instrumentation of the pulp chamber do not negatively impact fracture resistance, the subsequent decrease in fracture resistance observed following obturation and restoration remains concerning. In veterinary dentistry, endodontic treatment of discolored teeth typically occurs in the absence of severe periodontal disease, as in the case of teeth sustaining concussion injuries. These teeth suffer from pulpitis leading to pulp necrosis, without initial bacterial invasion observed histologically.^7^ Although the necrotic pulp may be sterile before an access is made in the pulp cavity, contamination occurs during endodontic treatment. Bacterial contamination of any remaining pulp could lead to bacterial microleakage through root canal obturation and sealing materials, warranting treatment of the pulp chamber.^48-53^ Therefore, the veterinary practitioner must choose between endodontic treatment of the root canal with or without treatment of the pulp chamber, as demonstrated in groups 1 and 2 of this study, respectively. Ultimately, further studies are required to determine the clinical success and appropriateness of each technique, and to establish best practices for endodontic treatment in dogs.

Several limitations were identified in this study, primarily due to the ex vivo design. When evaluating the force application, the mechanical load applied to each tooth was concentrated at the incisal surface, the weakest part of the crown. Consequently, the proportion of complicated crown fractures under-represented clinical observations, since most tooth fractures resulted in uncomplicated crown fracture in the incisal third of the crown. Distributing the force application over a wider surface area would more accurately represent clinical scenarios, however, variations in tooth size coupled with the complex shape of dog teeth, limit mechanical testing in this manner. For this reason, applying forces to the tip of the tooth crown remains the most practical method for ex vivo fracture testing in dog canine teeth.

When evaluating the angle of force application, a 45° angle disto-occlusal to the long axis of the crown was used. This angle was selected based on previous biomechanical studies performed on dog canine teeth and to mimic compressive and shear forces that occur during mastication and pulling, respectively.^20,21^ However, it is unknown whether this angle represents the most common clinical situation resulting in failure. For example, dogs exhibiting cage-biting behavior may apply excessive shear forces by pulling, which may be more accurately represented by a force application perpendicular to the long axis of the tooth in a disto-mesial direction. Although there is a previously published study using ex vivo analysis in determining characteristic fracture patterns related to the force direction applied to dog canine teeth,^21^ there are no studies evaluating the most common fracture direction patterns seen clinically, nor whether significant differences exist between varying force application angles on the fracture resistance of dog canine teeth. Therefore, future studies investigating these questions would serve to optimize ex vivo study design in dogs, with the goal of more accurately representing clinical behaviors and conditions.

The fracture classification system used in this study was adapted from AVDC nomenclature and selected for its simplicity. Based on this adaptation of the AVDC nomenclature of complicated versus uncomplicated tooth fracture,^29^ pulp exposure resulting in a compromised coronal seal was used to define a complicated fracture. For example, any fracture in the middle or gingival third of the crown of group 1 teeth would be defined as a complicated crown fracture due to pulp exposure. However, most crown fractures in group 2 teeth would be classified as uncomplicated crown fractures regardless of location because of the presence of obturation materials within the pulp chamber which maintained a coronal seal. Although this definition did not accurately compare fracture location between or within groups, it consistently defined the presence or absence of a compromised coronal seal which is an important factor in determining the need for endodontic retreatment. In addition, 13/17 (76.5%) teeth with pulp chamber obturation and restoration sustained crown fractures within the composite restoration, and 25/30 (83.3%) of group 1 and 4 teeth sustained uncomplicated crown fracture, revealing that most fractures were superficial and within a few millimeters of the incisal tip. Therefore, this fracture classification system was deemed appropriate for the purposes of this study given the clinical applicability and similar fracture locations between samples.

Although the modified AVDC fracture classification adequately described the fracture types observed in this study, future studies may wish to include the severity of tooth loss sustained. This would be important clinically, as it may change the viability of retreatment options and overall tooth prognosis. Examples include oblique complicated crown/root fractures which may be salvaged with a combination of endodontic and periodontal surgery, or in cases of teeth with prosthodontic crowns where tooth injury may result in unsalvageable fracture necessitating tooth extraction. Although previous studies describe more detailed classification systems in a veterinary context,^2,21^ they apply to intact teeth. Therefore, future studies in veterinary endodontics may wish to adapt systems utilized in human endodontics,^33,54,55^ where complications arising from various endodontic treatments are documented by including involvement of restoration and obturation materials. This in turn would provide insight into the benefits and risks of various endodontic treatment options, guiding the veterinary dentist in making more informed treatment and retreatment decisions.

In conclusion, although conservative access and instrumentation appear to minimally impact the fracture resistance of endodontically treated canine teeth in dogs, the cause and clinical importance of tooth weakening secondary to pulp chamber RMGIC obturation and incisal access composite restoration observed in group 2 teeth remains in question. Even so, this technique appears to restore tooth stiffness and may have protective value in cases of a subsequent tooth fracture, by maintaining a coronal seal. In addition, it also allows for more complete instrumentation and disinfection of intact teeth. These observations carry important clinical implications for veterinary patients, where tooth fracture or inadequate disinfection may compromise endodontic treatment success, and where delayed diagnosis and non-compliance with follow-up are a common clinical occurrence.

## Materials

- R Version 4.0.3, R Core Team, R Foundation for Statistical Computing, Vienna, Austria
- RC Prep, Premier Dental, Plymouth Meeting, PA, USA
- EDTA 17% Solution, Pulpdent, Watertown, MA, USA
- GuttaFlow2, Coltene, Akron, Ohio, USA
- Parallax Veterinary Gutta Percha Points, Shipps Dental & Specialty Products, Marana, AZ, USA
- Ionoseal, Voco GmbH, Cuxhaven, Germany
- Bond-1, Pentron, Wallingford, CT, USA
- ENA HRi Biofunction, Micerium, Avegno, Italy
- Sledgehammer Self Cure Powder & Liquid, Keystone Industries, Gibbstown, NJ, USA
- G90 Screw Action Grip, TestResources, Shakopee, MN, USA
- MTS Insight 50 EL (model # 820.050-EL), MTS Systems Corporation, Eden Prairie, MN, USA
- 1 kN load cell (model #569327-02), MTS Systems Corporation, Eden Prairie, MN, USA
- Image J, National Institute of Health, Bethesda, MD, USA
- MTS TestSuite TW Elite, MTS Systems Corporation, Eden Prairie, MN, USA
